# Advances in Precision Diagnostics and Personalized Therapeutics for Prostate Cancer: An Integrated Precision Continuum from Risk-Adapted Detection to Biomarker-Directed Therapy and Dynamic Monitoring

**DOI:** 10.3390/cancers18121909

**Published:** 2026-06-11

**Authors:** Takahide Noro, Takanobu Utsumi, Rino Ikeda, Tatsuharu Sugimoto, Naoki Ishitsuka, Yodai Kadono, Yuta Suzuki, Shota Iijima, Yuka Sugizaki, Takatoshi Somoto, Ryo Oka, Takumi Endo, Naoto Kamiya, Hiroyoshi Suzuki

**Affiliations:** 1 Department of Urology, Toho University Sakura Medical Center, Sakura 285-8741, Japan; takahide.noro@med.toho-u.ac.jp (T.N.); naoki.ishitsuka@med.toho-u.ac.jp (N.I.); yuta.suzuki@med.toho-u.ac.jp (Y.S.); shouta.iijima@med.toho-u.ac.jp (S.I.); yuuka.kizuki@med.toho-u.ac.jp (Y.S.); takatoshi.soumoto@med.toho-u.ac.jp (T.S.); ryou.oka@med.toho-u.ac.jp (R.O.); takumi.endou@med.toho-u.ac.jp (T.E.); naoto.kamiya@med.toho-u.ac.jp (N.K.); hiroyoshi.suzuki@med.toho-u.ac.jp (H.S.); 2 Department of Urology, Toho University Graduate School of Medicine, Tokyo 143-8540, Japan; tatsuharu.sugimoto@med.toho-u.ac.jp (T.S.); yodai.kadono@med.toho-u.ac.jp (Y.K.); 3 Department of Urology, Seirei Sakura Citizen Hospital, Sakura 285-8765, Japan; 4 Department of Urology, Mihama Hospital, Chiba 261-0013, Japan

**Keywords:** prostate cancer, precision medicine, risk-adapted detection, magnetic resonance imaging, PSMA PET, germline testing, tumor profiling, liquid biopsy, artificial intelligence, implementation science

## Abstract

Precision medicine in prostate cancer (PCa) is increasingly best understood as a continuum linking risk-adapted detection, multimodal diagnosis and phenotyping, and implementation-ready decision pathways. Contemporary clinical guidelines emphasize structured diagnostic strategies, appropriate use of advanced imaging, and selective deployment of biomarkers when results can alter management. Upstream risk enrichment using polygenic risk scores and multivariable prediction models may improve the yield of clinically significant disease while mitigating harms related to overdiagnosis. At the point of suspicion, magnetic resonance imaging-first pathways and reflex biomarker testing provide practical tools to reduce unnecessary biopsy while maintaining safeguards for the detection of clinically important disease. Beyond diagnosis, prostate-specific membrane antigen positron emission tomography refines disease-state phenotyping in initial staging, biochemical recurrence, and limited-burden presentations, while standardized acquisition and reporting improve reproducibility and multidisciplinary communication. Germline and tumor-based molecular profiling should be operationalized as a longitudinal care process with clear consent, turnaround targets, and test-to-action rules that define what each result enables at specific decision nodes. Finally, longitudinal monitoring approaches, including liquid biopsy and artificial intelligence-enabled pathology, are evolving rapidly and require transparent reporting and rigorous risk-of-bias appraisal before broad clinical adoption. This narrative review synthesizes key evidence across the precision continuum and outlines a decision-node-based, test-to-action framework for maximizing clinical benefit, maintaining quality, and ensuring equitable access.

## Simple Summary

Precision medicine in prostate cancer is often equated with matching treatment to tumor genetics. In practice, however, personalized care begins earlier, with risk-adapted screening, optimized diagnostic pathways, advanced imaging, molecular testing, and longitudinal monitoring. This review proposes an integrated precision continuum for prostate cancer care. The framework links each diagnostic or biomarker test to a specific clinical decision, including whether to perform biopsy, how to define disease extent, when to perform genetic testing, and how to monitor treatment resistance. We also emphasize that precision tools should be implemented with clear ownership, timely reporting, quality assurance, and attention to equitable access. This approach may help clinicians use the right test at the right time, avoid unnecessary procedures, and reduce unwarranted variation in care.

## 1. Introduction

Precision medicine in prostate cancer (PCa) is often framed narrowly as targeted therapy matched to tumor genomics. In clinical oncology, however, precision is a longitudinal process that begins upstream and requires coordination across multiple decision points. Contemporary guidelines increasingly reflect this concept by providing state-specific algorithms for localized and advanced disease and by emphasizing the roles of biomarkers, imaging, and prior treatment exposure in decision-making [1,2,3,4]. In early detection, guideline emphasis has shifted toward shared decision-making, improved biopsy strategies, and selective incorporation of magnetic resonance imaging (MRI) and biomarkers when results can meaningfully influence management [5].

A continuum framework helps prevent two recurrent failure modes. First, diagnostic innovation may increase complexity without improving outcomes if it is not linked to explicit decision rules. Second, innovation may be applied indiscriminately when biomarker and disease-state definitions lack sufficient precision, increasing cost, uncertainty, and patient burden without proportional benefit. The precision continuum can be conceptualized across four interconnected layers: (i) risk-adapted detection; (ii) multimodal diagnosis and phenotyping; (iii) test-to-action interfaces for molecular and imaging results; and (iv) dynamic monitoring and adaptation. The central implementation principle is clear: each test should be ordered only when it has the potential to change what clinicians and patients do next. The novelty of this review lies not in describing each precision tool in isolation, but in integrating these tools into a decision-node-based, test-to-action framework across the full prostate cancer continuum. In this framework, risk stratification, imaging, molecular testing, treatment selection, and monitoring are evaluated according to whether they change a defined clinical action at a specific disease state.

This article was designed as a narrative review rather than a systematic review or meta-analysis. We searched PubMed/MEDLINE and relevant guideline documents for English-language articles addressing precision diagnostics, risk-adapted detection, imaging, molecular profiling, biomarker-directed therapy, and longitudinal monitoring in prostate cancer, with a primary focus on literature published from January 2014 to May 2026. Search terms included “prostate cancer,” “precision medicine,” “polygenic risk score,” “MRI,” “PSMA PET,” “urine biomarker,” “blood biomarker,” “germline testing,” “somatic testing,” “HRR,” “DDR,” “PARP inhibitor,” “radioligand therapy,” “PTEN,” “AKT inhibitor,” “ctDNA,” “AR-V7,” “artificial intelligence,” and “digital pathology.” Priority was given to clinical practice guidelines, randomized trials, prospective diagnostic or biomarker studies, major validation studies, regulatory updates, and high-quality contemporary reviews. Articles were selected according to their relevance to discrete clinical decision nodes within the proposed precision continuum. Because this was not a systematic review, no formal PRISMA flow diagram, meta-analysis, or structured risk-of-bias assessment was performed.

**Figure 1:**
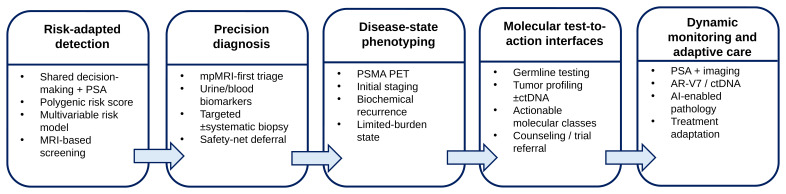
Integrated precision continuum for prostate cancer care.

This integrated precision continuum is summarized in Figure 1.

This schematic illustrates precision medicine as a longitudinal continuum from risk-adapted detection to precision diagnosis, disease-state phenotyping, molecular test-to-action interfaces, and dynamic monitoring/adaptive care. The implementation layer highlights operational requirements for safe deployment, including ownership, turnaround targets, structured reporting, multidisciplinary review, quality audit, and equity/access safeguards. The figure is intended to show the overall architecture of the proposed framework rather than a detailed clinical algorithm.

**Abbreviations:** AI, artificial intelligence; AR-V7, androgen receptor splice variant 7; ctDNA, circulating tumor DNA; mpMRI, multiparametric magnetic resonance imaging; MRI, magnetic resonance imaging; PRS, polygenic risk score; PSA, prostate-specific antigen; PSMA PET, prostate-specific membrane antigen positron emission tomography.

## 2. Precision Prevention and Risk-Adapted Early Detection

### 2.1. Polygenic Risk Score-Enriched Screening

Polygenic risk score (PRS) models derived from common germline variants may enrich screening populations for clinically significant PCa. In BARCODE1, men in the highest PRS decile were invited to undergo MRI and transperineal biopsy irrespective of prostate-specific antigen (PSA) level, and a substantial proportion of intermediate- or higher-risk cancers were detected, including cancers that would not have been captured by PSA-threshold pathways [6]. This study supports PRS as an upstream enrichment strategy that can increase diagnostic yield for clinically significant disease.

From an implementation standpoint, PRS-based screening does not replace PSA, MRI, or clinical judgment; rather, it reorganizes their use. PRS may help identify individuals who should enter more intensive imaging-based screening programs and those who may remain within less intensive surveillance schedules. Key challenges include ancestry calibration and counseling frameworks that translate probabilistic inherited risk into patient-centered decisions, including the possibility of increased anxiety and downstream testing.

A major implementation challenge is ancestry-related performance. Many prostate cancer PRS models have been developed and validated predominantly in European-ancestry populations, and discrimination and calibration may be attenuated in underrepresented populations. In an evaluation of PRS construction approaches among men of African and European ancestry, Darst et al. demonstrated ancestry-related differences in model performance, underscoring the need for ancestry-aware validation and recalibration before broad implementation [7]. Therefore, PRS should be used as an adjunct to shared decision-making and conventional clinical risk assessment rather than as a stand-alone screening determinant.

### 2.2. Multivariable Risk Models Coupled with MRI (STHLM3-MRI)

The STHLM3-MRI trial provides a complementary strategy that integrates clinical variables and biomarkers to triage MRI and targeted biopsy. Compared with PSA-based pathways, risk-model approaches can preserve detection of clinically significant disease while reducing unnecessary procedures and overdetection of low-grade cancers [8]. Their value lies not only in diagnostic performance but also in operational efficiency, as fewer men proceed to MRI and biopsy without compromising detection of clinically important cancers.

### 2.3. MRI-Based Screening Rounds and Biopsy Avoidance

Population-based screening studies incorporating MRI have shown that MRI-targeted approaches can preserve detection of clinically significant disease while reducing detection of clinically insignificant cancers [9]. Longer-term outcomes from repeated screening rounds further suggest that a negative high-quality MRI may justify biopsy omission in appropriately selected men, thereby reducing harms related to overdiagnosis and unnecessary procedures [10]. This reinforces a core principle of precision care: MRI can function both as a detection amplifier and as a de-escalation gatekeeper, depending on how it is integrated into a structured pathway.

## 3. Precision Diagnosis at the Point of Suspicion

### 3.1. MRI as a Pre-Biopsy Triage Tool (PROMIS) and MRI-First Diagnostic Pathways (PRECISION)

PROMIS demonstrated that multiparametric MRI (mpMRI) has high sensitivity for clinically significant PCa and can function as a triage test before initial biopsy, allowing a meaningful proportion of men to avoid unnecessary biopsy while improving detection of clinically significant disease when biopsy is directed by MRI findings [11]. This evidence provides the diagnostic foundation for MRI-first pathways.

PRECISION subsequently operationalized this concept in a randomized design, showing that an MRI pathway, comprising targeted biopsy for MRI-positive lesions and biopsy omission for MRI-negative men, can increase detection of clinically significant cancer and reduce detection of clinically insignificant cancer compared with systematic transrectal ultrasonography-guided biopsy [12]. Together, PROMIS and PRECISION support MRI as the central diagnostic modality around which reflex biomarkers, biopsy technique, and follow-up strategies may be structured.

However, MRI-first pathways should not be interpreted as eliminating the need for systematic biopsy or structured follow-up in all patients. In a large comparison of MRI-targeted, systematic, and combined biopsy, combined biopsy increased cancer detection compared with either approach alone, whereas MRI-targeted biopsy alone would have missed or misclassified a subset of clinically significant cancers [13]. Thus, mpMRI should be positioned as a triage and risk-refinement tool rather than as a stand-alone rule-out test.

### 3.2. Urine-Based Transcriptomics and Exosome Biomarkers

Urine-based assays provide noninvasive risk refinement and are particularly valuable in PSA gray-zone presentations, equivocal MRI findings, or situations in which patients wish to avoid biopsy. Development and validation of an 18-gene urine assay for high-grade PCa supported biopsy-reduction strategies at sensitivity thresholds designed to preserve detection of higher-grade disease [14]. Exosome-based urine testing has also shown clinical utility in real-world practice by influencing biopsy decision-making in men considering an initial biopsy [15]. Validation of additional urine exosomal ribonucleic acid expression approaches further supports the feasibility of pre-biopsy risk assessment across clinical settings [16].

A practical integration model uses urine biomarkers to resolve uncertainty rather than compete with MRI. Urine tests may help triage MRI demand when access is limited, support biopsy deferral after negative or equivocal MRI in men without high-risk features, or enrich biopsy yield by prioritizing men with high-risk biomarker profiles. The optimal pathway depends on local MRI availability, biopsy technique, patient preferences, and the clinical threshold for missing clinically significant disease.

### 3.3. Blood-Based and Other Reflex Tests in the MRI Era

Blood-based reflex tests can complement MRI-based strategies by improving specificity and reducing unnecessary biopsy. A real-world evaluation of the 4Kscore test in men undergoing MRI showed that the 4Kscore is associated with biopsy decision-making and improves predictive performance for clinically significant disease when combined with MRI [17]. These findings support pragmatic use of blood-based biomarkers as decision aids when MRI findings are equivocal or when clinical suspicion persists despite imaging.

For SelectMDx, a meta-analysis summarized diagnostic accuracy for clinically significant PCa and demonstrated moderate-to-good performance with substantial between-study heterogeneity, underscoring the need for high-quality prospective studies and careful positioning within diagnostic pathways [18]. In practice, these assays should be implemented with explicit action thresholds and with recognition that performance may vary by population, pretest probability, MRI use, and biopsy reference standard.

### 3.4. Tissue Biomarkers and Localized Decision Support

American Society of Clinical Oncology guidance supports selective use of molecular biomarkers in localized PCa when results can alter management, including active surveillance selection and decisions regarding adjuvant versus early salvage radiotherapy after prostatectomy [19]. This framework aligns with the continuum principle: biomarker testing should be anchored to a specific decision node rather than used as routine adjunctive testing without a clear clinical consequence.

## 4. Precision Staging and Disease-State Phenotyping: PSMA PET as a Strategy-Changing Modality

### 4.1. Initial Staging: proPSMA

The proPSMA study demonstrated that prostate-specific membrane antigen positron emission tomography (PSMA PET) improves staging accuracy compared with conventional imaging in high-risk PCa and leads to more frequent management changes [20]. Within the continuum framework, improved staging matters because accurate disease-state classification can alter treatment intent, field design for definitive local therapy, and systemic intensification strategies.

### 4.2. Biochemical Recurrence: CONDOR and Actionability

In biochemical recurrence with negative or equivocal conventional imaging, CONDOR showed that 18F-DCFPyL PET/CT can localize disease and meaningfully alter intended management, supporting PSMA PET as a tool that converts biochemical uncertainty into actionable anatomic phenotypes [21]. Operationally, localization can change salvage radiotherapy field design, eligibility for metastasis-directed therapy, and multidisciplinary planning.

### 4.3. Standardization for Implementation: Procedure Standards and Structured Reporting

A critical prerequisite for scaling PSMA PET is the standardization of acquisition, interpretation, and reporting. The joint European Association of Nuclear Medicine/Society of Nuclear Medicine and Molecular Imaging procedure guideline provides updated standards for indications, acquisition, and interpretation across PSMA radioligands and clinical scenarios, thereby supporting consistent implementation in both research and routine practice [22]. Standardization reduces inter-center variability, improves comparability across trials, and facilitates reliable multidisciplinary communication.

PROMISE V2 extends standardization by providing a structured framework for describing disease distribution using an updated molecular imaging tumor-node-metastasis system and harmonized reporting elements that improve clarity across institutions and teams [23]. In this review, the primary value of structured reporting is to support consistent disease-state phenotyping and reduce communication gaps when imaging findings are used to guide diagnostic and local-management decisions [20,21,22,23].

### 4.4. Oligometastatic Prostate Cancer as a Precision “Bridge State”

Oligometastatic PCa is increasingly recognized as an intermediate phenotype between localized and polymetastatic disease, particularly in the PSMA PET era. A contemporary review highlights how advanced imaging has expanded detection of limited metastatic burden and describes evolving therapeutic strategies, including metastasis-directed therapy and hybrid systemic–local approaches [24]. This state is clinically important because greater diagnostic precision can alter therapeutic intent, often shifting management toward lesion ablation and structured follow-up.

Randomized trials provide an evidentiary foundation for metastasis-directed therapy (MDT) in selected settings. STOMP demonstrated longer androgen deprivation therapy (ADT)-free survival with MDT than with surveillance in oligorecurrent hormone-sensitive PCa [25]. ORIOLE showed improved short-term progression outcomes with stereotactic ablative radiotherapy versus observation in men with limited metastatic burden [26]. EXTEND demonstrated improved progression outcomes when MDT was added to intermittent hormone therapy, including eugonadal progression-free survival [27].

These data should be interpreted with appropriate caution. STOMP, ORIOLE, and EXTEND support the biological and clinical plausibility of MDT in selected oligometastatic or oligorecurrent settings, but the evidence base remains limited by modest sample sizes, phase II designs, relatively short follow-up, heterogeneous imaging definitions, and the absence of established overall survival benefit. In the PSMA PET era, stage migration further complicates comparisons with earlier trials and may redefine what is considered oligometastatic disease. Accordingly, MDT should be presented as an evidence-informed option for carefully selected patients rather than as a universally established standard.

## 5. Molecular Profiling and Hereditary Risk: The Diagnostic-to-Therapeutic Bridge

### 5.1. Germline DNA Repair Mutations and Testing Beyond Family History

A landmark analysis demonstrated that inherited deoxyribonucleic acid (DNA) repair gene mutations are present in a clinically meaningful proportion of men with metastatic PCa and are not reliably identified by age or family history alone [28]. This supports germline testing as both an intervention for familial risk assessment and a gateway to biomarker-directed clinical decisions.

### 5.2. Practical Implementation of Hereditary Precision Care

A practice-oriented review synthesizes indications for genetic testing, counseling, syndrome-specific surveillance, and therapeutic implications across hereditary urologic cancers, emphasizing workflow design, counseling capacity, selection of actionable gene panels, and equitable access [29]. Implementation is central because testing recommendations do not translate into patient benefit unless results are delivered, interpreted, and acted upon in a timely and standardized manner.

### 5.3. Somatic Testing and Actionable Classes

Somatic testing complements germline assessment by identifying actionable alterations, including homologous recombination repair (HRR) and DNA damage repair (DDR) gene alterations, microsatellite instability-high (MSI-H) or mismatch repair-deficient (dMMR) status, and phosphoinositide 3-kinase (PI3K)–AKT pathway activation, often through phosphatase and tensin homolog (PTEN) loss. Across the continuum, utility is greatest at defined transition points: diagnosis of metastatic disease, progression to metastatic castration-resistant prostate cancer, and scenarios in which a molecular result is required for referral, trial screening, or multidisciplinary review. A major implementation priority is to deploy tissue- and liquid-based modalities efficiently to minimize delays in treatment decision-making. When tissue is limited, circulating tumor DNA (ctDNA) can complement profiling, but reporting standards and clinically meaningful thresholds remain essential [30].

## 6. Test-to-Action Interfaces: Operationalizing Precision Without Redundant Therapeutic Detail

### 6.1. Why a Test-to-Action Focus Is Necessary

Precision care can fail even when high-quality tests are available. One common failure mode is testing without action: biomarkers are ordered, results return late or in non-actionable formats, and management proceeds unchanged. Another is action without standards: tests are used inconsistently across sites, with variable acquisition, reporting, and interpretation that erode reproducibility. The goal of this section is to define operational interfaces, including where a test is ordered, how it is reported, and which next-step decisions it enables, so that diagnostic and molecular information reliably changes management at specific decision nodes across the continuum [1,2,3,4,5].

**Figure 2:**
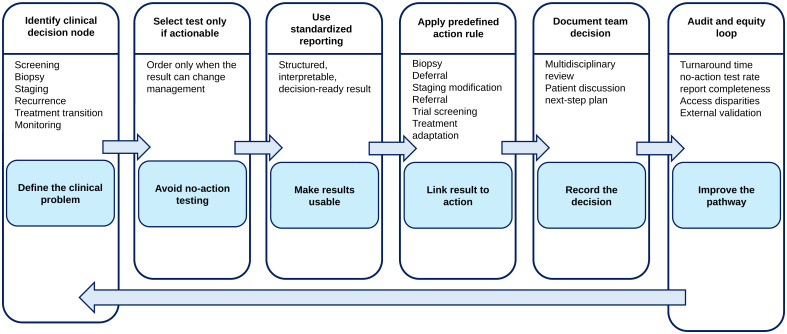
Test-to-action workflow for implementing precision tools in prostate cancer care.

A practical test-to-action workflow for linking precision diagnostics to predefined clinical actions is shown in Figure 2.

This workflow emphasizes that precision tests should be ordered only when their results can inform a predefined clinical action. The process begins with identification of a specific clinical decision node, followed by actionable test selection, standardized reporting, application of a predefined action rule, documentation of the multidisciplinary and patient-centered decision, and continuous audit. The audit loop monitors turnaround time, no-action test rates, report completeness, access disparities, and bias appraisal.

### 6.2. Decision Nodes for Inherited and Tumor Testing

Inherited and tumor alterations influence clinical decisions only if testing is performed early enough to be clinically usable and if consent and counseling pathways are embedded in routine workflows. Evidence indicates that inherited DNA repair gene mutations in metastatic PCa are not reliably captured by classic filters such as family history [28]. Practice-oriented frameworks emphasize that genetic testing is a longitudinal process that includes consent, referral pathways, result disclosure, and cascade considerations, rather than a single laboratory event [29].

Operationally, pragmatic decision nodes include: (i) confirmation of metastatic disease; (ii) transition points at which tissue may be scarce or turnaround time becomes rate-limiting; and (iii) situations in which local pathways require biomarker documentation for referral, trial screening, or multidisciplinary review. When archived tissue is inadequate, liquid biopsy can complement tissue testing, particularly for actionable genotyping, but its use should be paired with clear reporting standards and clinically meaningful thresholds [30].

### 6.3. Reporting Actionable Classes Rather than Listing Tests

To avoid over-testing and under-action, results should be reported in a format that maps directly to a decision. Clinically, this often means classifying findings into a small number of actionable categories that remain stable across institutions: HRR/DDR alterations, MSI-H/dMMR status, PTEN loss or PI3K–AKT pathway activation, and imaging-defined phenotypes relevant to disease-state classification.

Actionable does not mean automatic: assay limitations, tissue adequacy, and gene-level heterogeneity can influence confidence and should be communicated in the report. Professional guidance in localized disease supports selective biomarker use only when a result can change management, providing a generalizable template for limiting unnecessary testing across the continuum [19]. At the metastatic end of the continuum, guideline algorithms now explicitly incorporate biomarker-informed, mechanism-distinct options and emphasize the importance of early, workflow-ready testing and clinically justified retesting, reinforcing that testing is only as valuable as the action it enables [1,2,4].

### 6.4. Imaging-to-Action: PSMA PET as Disease-State Phenotyping

PSMA PET changes clinical management primarily by refining disease-state phenotyping and enabling anatomically informed plans in scenarios where conventional imaging is insufficient. In high-risk initial staging, PSMA PET improves staging accuracy and increases the likelihood that treatment intent reflects the true disease distribution [20]. In biochemical recurrence, PSMA PET can localize disease when conventional imaging is negative or equivocal, converting biochemical uncertainty into actionable anatomic patterns that influence salvage planning [21]. In limited-burden presentations, PSMA PET supports recognition of oligometastatic or oligorecurrent phenotypes and informs metastasis-directed strategies tested in randomized trials [24,25,26,27].

Because PSMA PET increasingly determines downstream decisions, standardized acquisition and reporting are essential. Procedure standards and structured reporting frameworks reduce inter-center variability and support consistent multidisciplinary communication [22,23]. In this manuscript, PSMA PET is discussed as a state-defining tool and an implementation challenge; detailed therapeutic delivery aspects of radioligand therapy are intentionally addressed elsewhere to avoid redundancy.

### 6.5. Workflow Design: Ownership, Turnaround Targets, and Measurable Outcomes

A test-to-action design requires explicit ownership and turnaround targets. Germline testing workflows should specify who obtains consent, who discloses results, and how cascade testing is offered, consistent with practice-oriented recommendations [29]. Tumor and liquid profiling workflows should specify specimen sources, minimum quality criteria, reporting formats, and timelines aligned with clinical decision points [30]. For imaging, local pathways should specify indications, acquisition standards, and how structured reports are embedded in multidisciplinary reviews [20,21,22,23].

A pragmatic quality metric is not the number of tests ordered, but the proportion of tests that change a documented next-step decision, such as referral, plan modification, or trial screening. When test results do not alter management, teams should review whether testing was ordered at an appropriate decision node, whether reporting was interpretable, and whether access barriers prevented action.

Key decision nodes, corresponding tests, intended actions, and implementation guardrails are summarized in Table 1.

### 6.6. Monitoring and AI as Implementation-Sensitive Layers

Monitoring tools and artificial intelligence (AI)-enabled decision support are attractive because they promise scalability, but their clinical value depends on transparent reporting and rigorous appraisal. Evidence suggests potential utility of circulating tumor cell-based biomarkers, such as androgen receptor splice variant 7 (AR-V7), and ctDNA for decision support in advanced disease, but implementation should remain linked to discrete decisions rather than continuous signal chasing [30,31,32]. Similarly, AI-enabled pathology tools are moving from development to validation [33,34], and reporting standards (TRIPOD+AI) and risk-of-bias tools (PROBAST+AI) provide necessary guardrails for trustworthy adoption [35,36]. In a precision continuum framework, these tools should be introduced only when they answer a defined clinical question, demonstrate reproducible performance in external validation, and have a plausible pathway to change management and improve patient-centered outcomes.

## 7. Precision Monitoring and Adaptive Care

### 7.1. AR-V7 in Circulating Tumor Cells

Detection of AR-V7 in circulating tumor cells has been associated with resistance to enzalutamide and abiraterone and with poorer outcomes on androgen receptor (AR) pathway inhibitor therapy, supporting AR-V7 as a clinically meaningful resistance phenotype [31]. Prospective multicenter validation further supports AR-V7 as independently associated with worse outcomes on hormonal therapies and suggests preserved taxane activity in some AR-V7-positive patients, reinforcing its potential value for decision support rather than as a purely prognostic label [32]. Within a continuum framework, AR-V7 is most valuable when it changes the next clinical action, such as mechanism switching, trial selection, or consideration of a non-AR-dependent strategy.

### 7.2. ctDNA: Genotyping, Clonal Evolution, and Monitoring

A contemporary review summarizes the roles of circulating tumor DNA (ctDNA) in PCa, emphasizing established utility in advanced disease for actionable genotyping when tissue is limited, as well as emerging roles in tracking clonal evolution and treatment response [30]. As ctDNA assays become more integrated into practice, standardization of pre-analytical variables, reporting, and clinically meaningful thresholds will determine whether ctDNA evolves from a supplementary assay into a routine longitudinal tool for adaptive decision-making.

## 8. AI and Digital Pathology: Scalable Decision Support Within the Continuum

### 8.1. Predicting Benefit from Treatment Intensification

An AI-based digital pathology biomarker has been developed and validated to predict benefit from long-term hormonal therapy combined with radiotherapy in high-risk PCa across multiple phase III trials, supporting benefit-based personalization of ADT duration rather than uniform intensification [33]. This addresses a common clinical dilemma: balancing oncologic benefit against the long-term morbidity associated with prolonged ADT exposure.

External validation of a multimodal AI-derived prognostic model in advanced PCa using STAMPEDE platform data suggests that baseline biopsy slides contain prognostic information capable of refining risk beyond traditional clinical stratification, with potential future implications for treatment selection [34]. Together, these studies suggest a role for AI as a scalable layer that can enhance decision fidelity, particularly when integrated with molecular and imaging biomarkers. Nevertheless, these AI studies should be viewed as promising but not yet fully deployment-ready. Both treatment-benefit prediction and prognostic AI pathology studies often rely on retrospective or post hoc ancillary biomarker analyses of phase III trial datasets. Although such datasets are valuable, they may introduce selection bias, may not fully represent real-world tissue processing and scanning variability, and do not by themselves establish prospective clinical utility. Before routine implementation, AI pathology tools require prospective validation, assessment across diverse practice settings, predefined clinical action thresholds, and transparent reporting of model development, calibration, external validation, and failure modes.

### 8.2. Ensuring Trustworthy AI Evidence: Reporting and Risk-of-Bias Standards

As AI-driven tools proliferate, evidence quality and transparency become integral to clinical adoption. TRIPOD+AI provides updated reporting guidance for prediction model studies using regression or machine learning methods and is intended to improve completeness, reproducibility, and interpretability of AI model reports [35]. The PROBAST+AI updates risk-of-bias and applicability assessment for prediction models using regression or AI methods, providing structured appraisal tools for researchers, reviewers, and guideline developers [36]. Incorporating these standards strengthens not only AI studies but also the broader precision continuum by improving confidence in decision-support claims and reducing the risk of deploying poorly validated models. In the context of the studies reviewed above, TRIPOD+AI and PROBAST+AI should be used not only as future reporting standards but also as interpretive guardrails. These frameworks help distinguish analytical validity and prognostic performance from true clinical utility, particularly when AI outputs are intended to influence treatment intensification or de-escalation.

## 9. Implementation: Linking Tests to Decisions, Preserving Quality, and Improving Equity

Implementation ultimately determines whether precision tools improve outcomes. The most effective strategy is to map each test to a clearly defined decision node. Risk enrichment using PRS or multivariable models should specify whether the result triggers MRI-based screening, alters screening intervals, or prompts genetic counseling [6,7,8]. MRI-first diagnosis should define how negative and equivocal MRI findings are managed and where reflex biomarkers should be incorporated [11,12,13,14,15,16,17,18]. PSMA PET should specify the clinical scenarios that justify its use and how standardized acquisition and reporting are maintained [20,21,22,23]. In metastatic disease, germline and tumor testing should be embedded in workflows that ensure predictable turnaround times, standardized reporting, and predefined test-to-action rules specifying how each result will alter the next clinical decision [28,29,30].

Salvage therapy after radical prostatectomy provides a concrete example in which coordinated imaging, risk stratification, and multidisciplinary decision-making are essential. The American Urological Association/American Society for Radiation Oncology/Society of Urologic Oncology salvage therapy guideline provides evidence- and consensus-based recommendations for nonmetastatic biochemical recurrence after prostatectomy and highlights the need for coordinated care in time-sensitive pathways [37]. Within the continuum framework, salvage pathways are an ideal setting in which to integrate refined imaging, pathology, and biomarkers because decisions are time-sensitive and the window for curative intent may narrow with delay.

A practical implementation checklist for building, measuring, and auditing a precision continuum program is provided in Table 2.

## 10. Limitations

This review has several limitations. First, it was designed as a narrative review rather than a systematic review or meta-analysis; therefore, the literature selection may be subject to selection bias despite our effort to prioritize guidelines, randomized trials, prospective studies, major validation studies, and contemporary high-quality reviews. Second, the strength of evidence varies substantially across the continuum. Some treatment strategies are supported by phase III randomized trials with survival endpoints, whereas several diagnostic biomarkers, liquid biopsy applications, oligometastatic MDT strategies, and AI-enabled pathology tools are supported primarily by diagnostic accuracy studies, phase II trials, retrospective analyses, or post hoc biomarker studies. Third, the implementation of precision tools depends on local resources, reimbursement systems, imaging and pathology infrastructure, molecular testing turnaround time, and access to genetic counseling. Finally, equity remains a central challenge, particularly for PRS, commercial biomarker assays, advanced imaging, radioligand therapy, and AI tools, all of which may perform differently or be less accessible across populations and healthcare systems.

## 11. Conclusions

Precision medicine in PCa is most effective when implemented as an end-to-end continuum rather than as a collection of isolated tests. Risk enrichment strategies, MRI-first diagnostic pathways, and reflex biomarkers can reduce unnecessary procedures while preserving detection of clinically significant disease. PSMA PET improves disease-state phenotyping in clinically consequential scenarios, and standardized acquisition and reporting frameworks support reproducibility and multidisciplinary communication. Molecular profiling should be operationalized as a longitudinal process with clear consent, turnaround targets, and explicit test-to-action rules so that results reliably change management when they matter. Emerging monitoring and AI-based tools must be integrated cautiously, guided by transparent reporting and rigorous bias appraisal to ensure that claimed clinical utility is trustworthy before broad deployment. Future studies should move beyond analytical performance and evaluate whether precision pathways improve patient-centered outcomes, reduce unnecessary procedures, shorten time to actionable decisions, and narrow disparities in access. Prospective implementation studies, external validation across diverse populations, and standardized reporting frameworks will be essential for translating precision tools into durable clinical benefit. The next frontier is implementation: measurable quality standards and equitable access that allow precision strategies to deliver real-world benefit.

## Figures and Tables

**Table 1:** Test-to-action map across key decision nodes.

Clinical Decision Area	Key Clinical Question	Practical Tool(s)	Action Enabled/Guardrail	Key Refs
Risk-adapted screening	Who should enter screening or intensified screening?	Shared decision-making, PSA, PRS, multivariable risk model	Defines screening entry, screening interval, and MRI-based escalation; PRS requires ancestry-aware validation and counseling	[1,3,5,6,7,8]
Pre-biopsy triage	Who needs biopsy, and who may safely defer it?	mpMRI-first pathway, targeted ± systematic biopsy, urine/blood biomarkers	Supports targeted biopsy, systematic biopsy when appropriate, or biopsy deferral with a safety-net plan; avoid using MRI as a stand-alone rule-out test	[11,12,13,14,15,16,17,18]
Localized disease decision support	Can a biomarker result alter localized treatment decisions?	Selective tissue biomarkers	May inform active surveillance selection or adjuvant versus early salvage discussions; avoid routine testing without a management consequence	[19]
Disease-state phenotyping	How should disease extent be defined?	PSMA PET with standardized acquisition and reporting	Refines initial staging, biochemical recurrence localization, salvage field design, and multidisciplinary planning	[20,21,22,23]
Limited-burden disease	Is the patient a candidate for MDT or a hybrid local–systemic approach?	PSMA PET, metastasis-directed therapy trial context, structured follow-up	Identifies selected oligorecurrent or oligometastatic candidates; acknowledge definition heterogeneity, phase II evidence, and lack of established overall survival benefit	[24,25,26,27]
Inherited and tumor profiling	Is there an actionable inherited or tumor alteration?	Germline testing, tumor profiling, ctDNA when tissue is limited	Enables familial risk assessment, cascade counseling, referral, trial screening, or treatment readiness; requires consent, tissue adequacy, and interpretable reporting	[28,29,30]
Resistance and longitudinal monitoring	Is there evidence of resistance or clonal evolution that should change management?	AR-V7, ctDNA, PSA, imaging	Supports treatment switching, trial selection, or continued monitoring; avoid continuous signal chasing without predefined action thresholds	[30,31,32]
AI-enabled decision support	Is the model sufficiently validated to support a clinical decision?	AI pathology tools, TRIPOD+AI, PROBAST+AI	Requires external validation, bias/applicability appraisal, workflow integration, and prospective clinical utility before broad deployment	[33,34,35,36]
Salvage pathway coordination	Can time-sensitive recurrence decisions be coordinated effectively?	PSMA PET, risk stratification, multidisciplinary review	Supports salvage planning after local therapy; requires timely decision-making and documentation of treatment intent	[21,22,23,37]

**Abbreviations:** AI, artificial intelligence; AR-V7, androgen receptor splice variant 7; ctDNA, circulating tumor DNA; MDT, metastasis-directed therapy; mpMRI, multiparametric magnetic resonance imaging; MRI, magnetic resonance imaging; PROBAST+AI, Prediction model Risk Of Bias ASsessment Tool plus Artificial Intelligence; PRS, polygenic risk score; PSA, prostate-specific antigen; PSMA PET, prostate-specific membrane antigen positron emission tomography; TRIPOD+AI, Transparent Reporting of a multivariable prediction model for Individual Prognosis Or Diagnosis plus Artificial Intelligence.

**Table 2:** Implementation checklist for a precision continuum program.

Implementation Domain	Minimum Requirement	Quality Metric	Equity/Access Safeguard
Pathway governance	Written decision-node map linking each test to a predefined clinical action	Proportion of tests with documented action change	Simplified pathway options for lower-resource settings
Imaging infrastructure	mpMRI and PSMA PET protocols with structured reporting standards	Structured-report completeness rate; compliance with MRI-negative follow-up plan	Referral network and triage rules to reduce unequal access to MRI or PSMA PET
Biomarker and genomic workflow	Predefined thresholds, consent pathway, specimen standards, and turnaround targets for germline, tumor, and ctDNA testing	Eligible-patient testing rate; adequate tissue rate; interpretable-report rate; turnaround time	Counseling access, coverage pathway, and liquid biopsy option when tissue is limited
Multidisciplinary and treatment documentation	Predefined action rules for biopsy deferral, salvage planning, MDT selection, trial referral, or treatment adaptation	Rate of documented multidisciplinary team decisions and next-step plans	Transparent eligibility criteria to avoid selective access to MDT or advanced testing
Monitoring governance	Action-trigger definitions for biomarker, imaging, ctDNA, AR-V7, or AI-derived changes	No-action test rate; actionability audit; avoidance of signal chasing	Avoid over-testing driven by availability or patient resources
AI evidence governance	TRIPOD+AI/PROBAST+AI-aligned review before implementation	Completion of bias, calibration, external validation, and applicability checklist	No routine deployment without external validation across relevant populations and practice settings
Continuous quality improvement	Regular audit of turnaround time, no-action testing, report completeness, access disparities, and patient-centered outcomes	Periodic pathway review and documented corrective actions	Community-site referral routes and monitoring for disparities in test access and downstream action

**Abbreviations:** AI, artificial intelligence; AR-V7, androgen receptor splice variant 7; ctDNA, circulating tumor DNA; MDT, metastasis-directed therapy; mpMRI, multiparametric magnetic resonance imaging; MRI, magnetic resonance imaging; PROBAST+AI, Prediction model Risk Of Bias ASsessment Tool plus Artificial Intelligence; PSMA PET, prostate-specific membrane antigen positron emission tomography; TRIPOD+AI, Transparent Reporting of a multivariable prediction model for Individual Prognosis Or Diagnosis plus Artificial Intelligence.

## Data Availability

No new data were created or analyzed in this study. Data sharing is not applicable to this article.

## Abbreviations

The following abbreviations are used in this manuscript:

ADT	androgen deprivation therapy
AI	artificial intelligence
AR	androgen receptor
AR-V7	androgen receptor splice variant 7
csPCa	clinically significant prostate cancer
ctDNA	circulating tumor DNA
DDR	DNA damage repair
dMMR	mismatch repair-deficient
DNA	deoxyribonucleic acid
HRR	homologous recombination repair
MDT	metastasis-directed therapy
mpMRI	multiparametric magnetic resonance imaging
MRI	magnetic resonance imaging
MSI-H	microsatellite instability-high
PCa	prostate cancer
PET	positron emission tomography
PET/CT	positron emission tomography-computed tomography
PI3K	phosphoinositide 3-kinase
PRS	polygenic risk score
PSA	prostate-specific antigen
PSMA PET	prostate-specific membrane antigen positron emission tomography
PTEN	phosphatase and tensin homolog
PROBAST+AI	Prediction model Risk Of Bias ASsessment Tool plus Artificial Intelligence
TRIPOD+AI	Transparent Reporting of a multivariable prediction model for Individual Prognosis Or Diagnosis plus Artificial Intelligence
